# The Contagion of Unethical Pro-organizational Behavior: From Leaders to Followers

**DOI:** 10.3389/fpsyg.2018.01102

**Published:** 2018-07-03

**Authors:** Yun Zhang, Bin He, Xu Sun

**Affiliations:** ^1^School of Management, Guangdong University of Technology, Guangzhou, China; ^2^School of Business, Wuzhou University, Wuzhou, China

**Keywords:** leader identification, unethical pro-organizational behavior, moral identity, social identity theory, contagion

## Abstract

Unethical pro-organizational behavior is a common phenomenon in businesses, and one that can cause great damage to them as well as to wider society. Although prior studies have investigated why individuals engage in unethical pro-organizational behavior, little research has been undertaken into why such behavior might be commonplace in organizations. The present study focuses on the downstream contagion of unethical pro-organizational behavior from leaders to followers. Drawing on social identity theory, we consider why leaders’ unethical pro-organizational behavior brings about corresponding behavior in their employees. Moreover, we predict that leader identification and moral identity will moderate this relationship. Using a time-lag study design, we collected a sample of 227 multisource time-lagged data with which to test our hypotheses. The results show that there is a significant positive relationship between leaders’ and employees’ unethical pro-organizational behavior, and that this relationship is stronger when employees have higher leader identification and lower moral identity levels. The theoretical and practical implications of our findings are discussed in this paper, as are the limitations of the study.

## Introduction

Unethical behavior in the workplace has been widely observed (Treviño et al., 2006), with many employees contending that their unethical activities serve to benefit the organization or its members (Umphress et al., 2010). “Unethical pro-organizational behavior” (hereafter, “UPB”) such as this is defined as “actions that are intended to promote the effective functioning of the organization or its members (e.g., leaders) and violate core societal values, norms, laws, or standards of proper conduct” (Umphress et al., 2010, p. 622), such as a tendency to “exaggerate the truth about one’s company’s products or services to customers and clients to benefit one’s company.” Such behavior appears to help the organization in the short term (Umphress et al., 2010), but comes at a high cost to the business in the long run (Cialdini et al., 2004; Dunlop and Lee, 2004). For example, Kobe Steel has admitted to falsification over past years relating to large quantities of some types of material, with its employees (including managers) in multiple outlets forging data for the economic benefit of the company (Ying, 2017). This scandal has gone on to have a huge impact on both the company and the wider Japanese steel industry.

Scholars are generally interested in why an individual might engage in UPB (Umphress and Bingham, 2011). In attempting to answer this question, prior studies have examined the impact of organizational (e.g., workplace exclusion; Thau et al., 2015), interpersonal (e.g., transformational leadership, ethical leadership; Miao et al., 2013; Effelsberg et al., 2014), and individualfactors (e.g., organizational identification) on UPB (Umphress et al., 2010; Chen et al., 2016), which has enabled us to have a better understanding of why employees engage in such behaviors. However, prior research has not explained why UPB is not a single case in the organization, but, rather, a widespread phenomenon (as was the case with Kobe Steel). Evidently, there is a contagion of UPB within organizations, yet the existing literature does not explain this phenomenon well. Consequently, our study, drawing on social identity theory (Ashforth and Mael, 1989), attempts to answer the question of why such unethical behavior (in the name of benefitting the organization) spreads from managers to subordinate staff members (Gino and Pierce, 2009; Gino et al., 2009a).

In this study, we also consider the question of when leaders’ UPB (hereafter, “LUPB”) might bring about employees’ UPB (“EUPB”). According to social identity theory, supervisors may influence their subordinates by affecting certain elements of a subordinate’s self-concept (Van Knippenberg et al., 2004). “Self-concept” in this context is taken to include the collective self, the relational self, and the personal self (Brewer and Gardner, 1996; Miao et al., 2013). Building on this logic, we suggest two factors that are likely to influence the strength of the positive relationship between LUPB and EUPB. First, we propose that an employee who has higher leader identification (i.e., the relational self; Van Knippenberg et al., 2004; Miao et al., 2013) will be more likely to act according to the values of their leaders, thereby increasing the chances that they will follow LUPB. Second, we posit that an employee with lower moral identity (i.e., the personal self; Aquino and Reed, 2002) is more likely to ignore moral principles and follow LUPB.

This study makes several contributions to the existing literature. First, we extend a theoretical framework regarding the spread of UPB, using social identity theory. Previous scholars have discussed the contagion of unethical behavior between co-workers based on self-categorization theory (Gino et al., 2009b) and norm-focus theory (Gino et al., 2009a), but without noting the role of self-concept. Therefore, we contribute to the UPB literature by constructing a theoretical framework based on social identity theory from the perspective of self-concept. Second, as stated earlier, prior research has not explained why UPB spreads in an organization. Exploring these contamination processes should elicit interesting insights into why and how UPB transmutes into a universal phenomenon across an organization. Our study is based on social identity and self-concept perspectives, which help to theoretically explain why subordinates will follow their leaders’ engagement in UPB, and is therefore one of the first to explain the contagion mechanism of UPB. Third, we advance the leadership literature by focusing on the negative effects of leader identification, whereas prior studies in this area have generally concentrated on its positive side (Pratt, 1998; Wing et al., 2018), largely neglecting its dark side. We contend that LUPB will have a stronger influence on EUPB when the followers have higher leader identification. Fourth, our study also investigates whether the strength of the relationship between LUPB and EUPB will differ between subordinates with different moral identity. Specifically, we propose that the relationship between LUPB and EUPB will be stronger when subordinates have lower moral identity. Finally, previous research has explored the contagion of unethical behavior either theoretically or experimentally (Gino et al., 2009a; Moore and Gino, 2013). The present study uses field survey data to advance previous research. Our research also contributes to the existing literature by answering the call for investigations into the spread of unethical behavior in the workplace (Treviño et al., 2014). The results of our study enable us to make practical suggestions, as well, for organizations on how best to control the spread of UPB.

## The Relationship Between Lupb and Eupb

Employees have been known to, for example, exaggerate the function of health products to gain profits for their company, or overstate sales to mislead consumers, or commit accounting fraud in order to protect their company and their managers (Ertugrul and Krishnan, 2011). In such cases, these employees will often argue that they take part in the unethical behavior for the benefit of the company, but not for themselves. This kind of unethical behavior is classified as UPB because it has the characteristics of being “pro-organization” and its purpose is to benefit the organization or its members. Unlike self-concern-related unethical behavior, UPB tends to benefit others in the organization. However, UPB can’t necessarily be separated from self-concern behavior because the actors may benefit themselves through benefitting the organization or its employees (Umphress and Bingham, 2011).

According to social identity theory, leaders can help individuals establish standards of “right” behavior in their organization (Graham et al., 2015). Correspondingly, when LUPB is observed, individuals tend to think that they should do the same in the organizational context, and convince themselves that such behavior is ethical (Gino et al., 2013). Similarly, observing leaders’ unethical behavior can change people’s estimates of the likelihood of being punished for carrying out comparable acts (Gino et al., 2009a), and cause them to recalculate the cost versus benefit of similar behavior (Becker, 1968). Indeed, unethical behavior can often appear to bring more benefits than ethical behavior (Gino et al., 2009a). Moreover, recent studies have shown that most people choose to engage in unethical behavior when the cost of engaging in such behavior decreases (Ayal and Gino, 2012). Thus, when LUPB is observed, individuals will recalculate the cost and benefits of UPB and tend to follow such behavior, thereby attaining more profits.

Additionally, observing LUPB can change an individual’s view of the morality of the current issue. The social categorization process has an impact on an individual’s moral perception such that the behavior exhibited by members of the organization is believed to be ethically acceptable, even if it is not (Gino et al., 2009a). Previous studies have shown that, when the categorization of a particular behavior is not clear, people tend to classify the behavior in a positive way in order to avoid negatively updating their moral self-image (Schweitzer and Hsee, 2002). UPB seems good for organizations even though it is not in line with social morality, so the categorization of UPB is not clear. Thus, when LUPB is observed, the individual’s moral judgment about such behavior may change and the UPB would be positively classified and considered an appropriate behavior in the organizational context. Accordingly, individuals will convince themselves that UPB is appropriate and be willing to follow suit (Gino et al., 2013).

Furthermore, organizations often develop norms that tolerate the violation of moral standards if it is beneficial to the organization (Moore and Gino, 2013). “Organizational norms” are the common behavioral expectations that people hold in a given organization (Marler et al., 2012), and encompass “descriptive norms” and “prohibitive norms” (Cialdini et al., 1990). Descriptive norms specify what an individual can do under certain circumstances, while prohibitive norms keep an individual from engaging in certain behaviors (Cialdini et al., 1990). By observing the behavior of others, people form their cognition of organizational norms and act in the way their organization seemingly expects (Rimal and Real, 2003). Observing the behavior of leaders also affects people’s understanding of the norms of organizational ethics (Gino et al., 2009a). Experimental studies have shown that, when people are surrounded by the unethical behavior of their peers, they are likely to imitate the behavior of these peers, because such behaviors demonstrate apparently appropriate organizational norms (Gino et al., 2009a). Therefore, when observing LUPB, subordinates will likely consider such behavior to be what the organization expects them to do, and they may then follow and engage in UPB themselves.

Taking these points together, we theorize that, when they observe LUPB, subordinates will recognize UPB as being allowed in the organization and will follow such behavior:

Hypothesis 1: LUPB is positively related to EUPB.

## The Moderating Role of Leader Identification

Drawing on social identity theory, several researchers have postulated that supervisors influence subordinates’ behaviors and attitudes by shaping their self-concept (Kark et al., 2003; Miao et al., 2013). An individual’s “self-concept” allows them to give meaning to their own memories and behaviors (Kihlstrom et al., 2003). It defines the “self” from three dimensions: (1) the personal self (i.e., personal characteristics), (2) the relational self (i.e., relationships with others), and (3) the collective self (i.e., membership of social groups; Brewer and Gardner, 1996). In this study, we apply two kinds of self-concept—the personal self and the relational self—as boundary conditions with which to construct an integrated framework for analyzing the influence of LUPB on EUPB.

“Leader identification” is “the extent to which a supervisor is included in the subordinate’s relational self” (Miao et al., 2013, p. 645). It is a generalized form of identification that has attracted a significant proportion of scholarly attention (Cooper and Thatcher, 2010). Individuals tend to identify with someone who can help them finish tasks or satisfy their psychosocial needs (Ashforth et al., 2008). In the organizational context, supervisors are the ones who create the relationships with subordinates (Sluss et al., 2012), and they are largely the ones who help employees to finish tasks and fulfill their psychosocial needs. Leader identification occurs when subordinates adopt supervisors’ attitudes, values, and behaviors in order to relate to a satisfying, self-defining relationship with supervisors (Becker, 1992). Employees’ leader identification may lead to behaviors such as helping and supporting supervisors (Sluss and Ashforth, 2007).

Subordinates with a high level of leader identification tend to internalize the interests, goals, and values of the leader, and are even willing to change their own values (e.g., beliefs, actions, etc.) so that they can attain further similarity with their leaders (Pratt, 1998). Accordingly, we expect that employees with high leader identification will more likely attend to follow leaders’ UPB in order to benefit their organizations. Further, when their supervisors engage in UPB, employees who have high identification with their immediate leaders will be more likely to consider this behavior as “right,” and even to follow such behavior. Miao et al. (2013) argued that subordinates with higher leader identification are more likely to be influenced and to have their views shaped by their leaders with respect to what is appropriate within the organization. From the analysis above, we posit that:

Hypothesis 2: Leader identification moderates the relationship between LUPB and EUPB, such that the relationship is stronger for employees with high leader identification than for those with low leader identification.

## The Moderating Role of Moral Identity

“Moral identity” is defined as “a self-conception organized around a set of moral traits” (Aquino and Reed, 2002, p. 1424). It reflects an individual’s internal moral standards, and the importance of the moral quality in the self-concept (Winterich et al., 2009). It also plays a self-regulating role in maintaining an individual’s moral self-image (Mulder and Aquino, 2013), whereby they will compare their present moral self-image and an idealistic moral self-image, and the gap between the two can lead to psychological pressure on the individual. This psychological pressure will, in turn, promote a consistency between an individual’s behavior and their internal morality. Thus, possessing high levels of moral identity will inhibit an individual from engaging in a variety of deviant behavior, such as lying, academic deception, and aggressive behavior (Aquino et al., 2009; Mulder and Aquino, 2013).

Therefore, we argue that an employee’s moral identity may play a negative moderating role on the relationship between LUPB and EUPB. That is, individual unethical behavior decisions are influenced not only by individual relational context, but also by personal moral characteristics (Kish-Gephart et al., 2010). In other words, if we only consider the influence of LUPB and leader identification, individuals will demonstrate moral deviations when making decisions. However, because people have different levels of moral identity, an individual’s ultimate behavior may also differ. It has been observed that individuals with a high level of moral identity will perceive a gap between current and ideal moral self-image and feel pressure, which means, in the context of the present study, that they would be less likely to follow their leaders to engage in UPB. Earlier studies have shown that morality increases personal obligation and responsibility to behave consistently with one’s moral concerns, as this is a core concept in an individual’s personal self (Matherne et al., 2012). Based on this, we expect:

Hypothesis 3: Moral identity moderates the relationship between LUPB and EUPB, such that the relationship is stronger for employees with low moral identity than for those with high moral identity.

## Methods

In order to ensure the reliability and validity of the study’s measurement tools, as well as the data collection, we used questionnaires that have been utilized and validated in earlier studies. In order to reduce common method bias, we collected multisource time-lagged data twice from full-time employees working in companies located in the Guangdong and Guangxi provinces of China. We asked the enterprises’ human resource departments to assist with the distribution of the questionnaires. The employees filled in the questionnaires daily, after work, and were told that the results of the surveys would be used for academic research only. We first collected data concerning the levels of LUPB, which was reported by managers. After 4 weeks, we then collected data in respect of EUPB, leader identification, and moral identity from the direct subordinates of the managers from the first time period. Both surveys also included a number of demographic control variables.

From the first time period, 278 valid questionnaires (84.06% response rate) were returned, and, from the second, 227 valid questionnaires, representing an effective return rate of the questionnaire of 81.65%. The average age of leaders was between 41 and 50 years old, and 41.90% were women. Leaders’ average number of years working in the same company was between 11 and 15 years. The average age of subordinates was between 23 and 30 years old, and 51.10% were women. Subordinates’ average years working in the same company were between 3 and 5 years.

### Measures

Except for the 7-point scale, ranging from 1 (“*strongly disagree*”) to 7 (“*strongly agree*”), that we used to measure the level of leader identification and UPB, most of the items were measured using a 5-point scale (ranging from 1 for “*strongly disagree*” to 5 for “*strongly agree*”).

#### Unethical Pro-organizational Behavior

We used the six-item measure developed by Umphress et al. (2010) to test LUPB (α = 0.80) and EUPB (α = 0.78). Sample items included “If my organization needed me to, I would withhold issuing a refund to a customer or client accidentally overcharged” and “If it would help my organization, I would exaggerate the truth about my company’s products or services to customers and clients.”

#### Leader Identification

A 7-item measure developed by Becker et al. (1996) was used to assess employees’ leader identification (α = 0.89). Sample items included “I feel a sense of ‘ownership’ for my supervisor” and “When someone praises my supervisor, it feels like a personal compliment.”

#### Moral Identity

A five-item measure was used, based on Aquino and Reed (2002) subscale of moral identity internalization (α = 0.88). Sample items were “I strongly desire to have these characteristics” and “Being someone who has these characteristics is an important part of who I am.”

#### Organizational Identification

Drawing on social identity theory, we determined that an individual’s organizational identification would affect any UPB (Umphress et al., 2010; Chen et al., 2016), so we controlled for this factor in our study. We used a six-item measure based on Mael and Ashforth (1992) six-item measure (α = 0.80), with a sample item being “My organization’s successes are my successes.”

#### Social Desirability

To control for an individual’s social desirability tendencies, as our items concerned a sensitive topic, we used the social desirability 10-item scale developed by Steenkamp et al. (2010) (α = 0.71), in which sample items included “I sometimes tell lies if I have to” and “When I hear people talking privately, I avoid listening.”

Finally, as the results from prior studies have suggested that participants’ gender (0 = female, 1 = male), age (1 = 18–22 years old, 2 = 23–30 years old, 3 = 31–40 years old, 4 = 41–50 years old, 5 = 51–60 years old), and years working in a company (1 = less than 2 years, 2 = 3–5 years, 3 = 6–10 years, 4 = 11–15 years, 5 = 16–20 years, 6 = 21–30 years, 7 = more than 31 years) would affect their willingness to engage in UPB (Thau et al., 2015; Chen et al., 2016), we controlled for these factors.

### Measurement Model

We performed a confirmatory factor analysis, and the results are presented in **Table 1**. The proposed four-factor structure (i.e., LUPB, organizational identification, moral identity, and EUPB) revealed an acceptable fit (Model 1): χ^2^(293) = 423.60, *p* < 0.001; CFI = 0.95; IFI = 0.95; RMSEA = 0.04. Conceivable alternative models with fewer factors (i.e., Models 2–5) did not fit our data.

**Table 1:** Comparison of Measurement Models.

Model	Descriptions	χ^2^	*df*	Δχ^2^	RMSEA	CFI	IFI
Model 1	Four factors: LUPB, LI, MI, EUPB	423.60	293		0.04	0.95	0.95
Model 2	Three factors: LUPB and LI were combined into one factor	794.21	296	370.61^∗∗∗^	0.09	0.79	0.79
Model 3	Three factors: LUPB and MI were combined into one factor	830.58	296	406.98^∗∗∗^	0.09	0.78	0.77
Model 4	Two factors: LUPB, MI, and LI were combined into one factor	1292.47	298	868.87^∗∗∗^	0.12	0.58	0.59
Model 5	One factor: LUPB, MI, LI, and EUPB were combined into one factor	1648.82	299	1225.22^∗∗∗^	0.14	0.43	0.44

*LUPB, leaders’ unethical pro-organizational behavior; LI, leader identification; MI, moral identity; EUPB, employees’ unethical pro-organizational behavior; RMSEA, root mean square error of approximation; CFI, comparative fit index; IFI, incremental fit index. ^∗∗∗^p < 0.001.*

## Results

The means, standard deviations, and correlations of variables are shown in **Table 2**. A significant positive effect was observed between LUPB and EUPB (*r* = 0.23, *p* < 0.01). **Table 3** presents the hierarchical regression analyses of the variables (we standardized the variables before analysis). As shown in Model 1 (**Table 3**), LUPB has a significant positive effect on EUPB (*b* = 0.16, *SE* = 0.07, *t* = 2.24, *p* < 0.05). This result supports Hypothesis 1.

**Table 2:** Means, standard deviations, and correlations.

Variable^a^	*M*	*SD*	1	2	3	4	5	6	7	8	9
(1) Gender^b^	0.49	0.50									
(2) Age	2.32	1.16	−0.08								
(3) Working year	2.41	1.49	0.20^∗∗^	0.81^∗∗^							
(4) SD	3.27	0.45	0.03	0.09	0.12	(0.71)					
(5) OI	2.94	0.59	−0.03	−0.01	−0.03	0.07	(0.80)				
(6) LUPB	3.64	1.12	−0.04	0.01	−0.09	0.07	0.38^∗∗^	(0.80)			
(7) LI	3.84	1.33	0.02	−0.07	−0.08	0.05	0.04	0.22^∗∗^	(0.89)		
(8) MI	2.75	1.16	−0.06	0.04	−0.03	0.05	−0.08	0.03	−0.29^∗∗^	(0.88)	
(9) EUPB	3.33	1.13	0.00	0.03	−0.02	−0.01	0.21^∗∗^	0.23^∗∗^	−0.01	−0.06	(0.78)

*N = 227. Coefficient alphas are given in parentheses on the diagonal. ^a^SD, socially desirable; OI, organizational identification; LUPB, leaders’ unethical pro-organizational behavior; LI, leader identification; MI, moral identity; EUPB, employees’ unethical pro-organizational behavior. ^b^Females were coded 0 and males were coded 1. ^∗∗^p < 0.01.*

**Table 3:** Results of hierarchical regression analyses with EUPB.

Variable^a^	EUPB^b^						
	Model 1		Model 2		Model 3	
	*B*	*SE*	*B*	*SE*	*B*	*SE*
Constant	1.97^∗∗^	0.65	2.19^∗∗^	0.64	2.09^∗∗^	0.66
Gender^c^	0.10	0.16	0.06	0.16	0.12	0.16
Age	0.12	0.12	0.12	0.12	0.16	0.12
Working year	−0.08	0.10	−0.09	0.09	−0.11	0.10
SD	−0.07	0.16	−0.05	0.16	−0.06	0.16
OI	0.30^∗^	0.13	0.25	0.13	0.29^∗^	0.13
LUPB	0.16^∗^	0.07	0.18^∗∗^	0.07	0.16^∗^	0.07
LI			−0.07	0.05		
LUPB × LI			0.21^∗∗^	0.05		
MI					−0.06	0.06
LUPB × MI					−0.16^∗∗^	0.06
F		2.97^∗∗^		4.77^∗∗^		3.39^∗∗^
*R*^2^		0.08		0.15		0.11
Δ*R*^2^				0.07^∗∗^		0.04^∗^

*N = 227. ^a^SD, socially desirable; OI, organizational identification; LUPB, leaders’ unethical pro-organizational behavior; LI, leader identification; MI, moral identity. ^b^EUPB, employees’ unethical pro-organizational behavior. ^c^Females were coded 1 and males were coded 0. ^∗^p < 0.05. ^∗∗^p < 0.01.*

**FIGURE 1:**
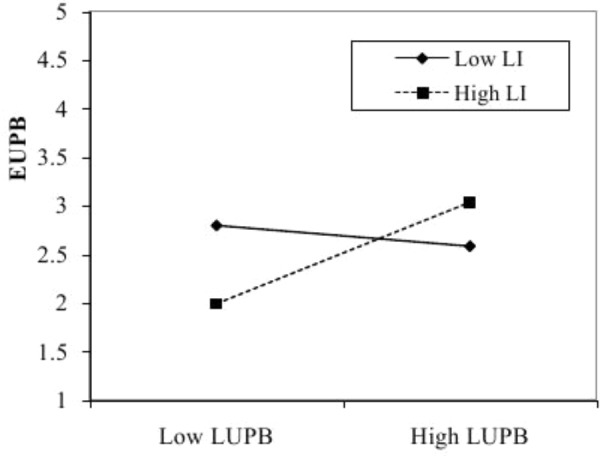
Interaction between LUPB and leader identification on EUPB. LUPB, leaders’ unethical pro-organizational behavior. EUPB, employees’ unethical pro-organizational behavior. LI, leader identification.

The interaction between LUPB and leader identification is significant (see **Table 3**, Model 2), *b* = 0.21, *SE* = 0.05, *t* = 4.26, *p* < 0.01. Additionally, we analyzed simple slopes of leader identification at ±1 *SD* of leader identification (**Figure 1**) (Aiken and West, 1994). The effect of LUPB on EUPB is significant and positive when leader identification was high (+1 *SD*) [simple slope = 0.39, *t*(227) = 4.71, *p* < 0.01], but non-significant when leader identification was low (−1 *SD*) [simple slope = −0.03, *t*(227) = −0.31, *p* = 0.76]. These results support Hypothesis 2.

**FIGURE 2:**
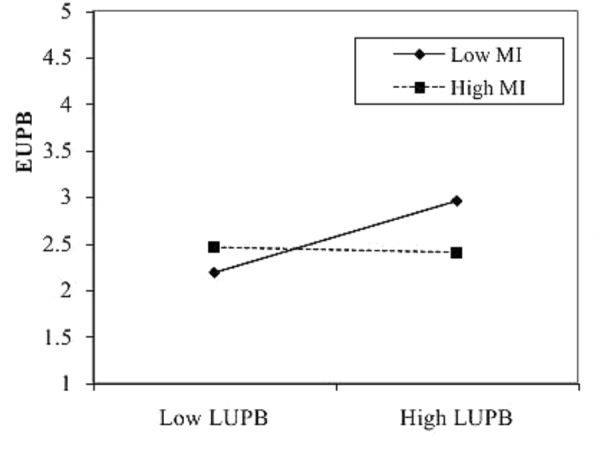
Interaction between LUPB and moral identity on EUPB. LUPB, leaders’ unethical pro-organizational behavior. EUPB, employees’ unethical pro-organizational behavior; MI, moral identity.

The interaction between LUPB and moral identity is significant (see **Table 3**, Model 3), *b* = −0.16, *SE* = 0.06, *t* = −2.82, *p* < 0.01. Additionally, we analyzed simple slopes of moral identity at ±1 *SD* (**Figure 2**). The moderation effect of moral identity is significant when it was low (−1 *SD*) [*b* = 0.32, *t*(227) = 3.38, *p* < 0.01], but non-significant when moral identity was high (+1 *SD*) [*b* = 0.00, *t*(227) = 0.01, *p* = 0.99]. This suggests that, when moral identity is low, LUPB has a significant positive effect on EUPB. Conversely, when moral identity is high, an individual’s willingness to follow her/his leader and engage in UPB is reduced. These results support Hypothesis 3.

## Discussion

In this study, we developed and tested a model that explicates why and when employees follow leaders to engage in UPB. Results from a field study provided empirical evidence that LUPB is related to EUPB, and that the effect of LUPB on EUPB is stronger when employees have higher leader identification and lower moral identity levels. We discuss the implications of these findings, as well as the limitations of and recommended future research directions from the present study, below.

### Theoretical Implications

Our research provides several contributions to the current theory. First, an innovative strand of the present work is our use of social identity theory to explain the spread of UPB, in contrast to past research that has described the contagion of unethical behavior between peers through reference to self-categorization and norm-focus theories (Gino et al., 2009a,b). Specifically, our research, based on social identity theory, proposes a research framework from the perspective of self-concept that fully explicates why and when UPB would spread from leaders to employees. Our analysis indicates that self-concept may play a vital role in the contagion of UPB between supervisors and subordinates, and thereby provides a theoretical explanation of the spread of unethical behavior from the perspective of self-concept.

The second important contribution of this study is that it is the first to explain the downstream contagion mechanism of UPB from leaders to employees. As noted above, some organizations experience an out-of-control proliferation of UPB while others do not (Peterson, 2004), and the reason for this difference is not yet clear (Sims and Brinkmann, 2002; Treviño et al., 2014). Our findings suggest that, when individuals observe LUPB, they may follow the behavior because LUPB alters their estimations regarding the likelihood of being punished for engaging in similar behaviors by recalculating the cost–benefit analysis of similar behavior (Gino et al., 2009a). Moreover, LUPB likely changes an employee’s view of similar behavioral categorization (Schweitzer and Hsee, 2002), and influences their understanding of organizational norms (Gino et al., 2009a). Thus, our research provides some explanation of how and why UPB spreads in an organization and goes on to become a universal phenomenon in the firm.

Third, we contribute to the identification literature by focusing on the negative effects of leader identification. As mentioned before, leader identification is mostly considered to be good for organizations (Wing et al., 2018). Conversely, our study’s results suggest that employees who have higher leader identification are more likely to take part in the same behavior as their supervisors blindly, even ignoring the ethical issues involved, because they have higher recognition with respect to their supervisors (Miao et al., 2013). By demonstrating that high leader identification does not necessarily result in positive consequences for the organization, our findings reveal the dark side of leader identification.

Fourth, our research advances current research in unethical behavior, in that we consider the boundary conditions of a top–down contagion. Prior research has mostly focused on the direct effect of the spread of unethical behavior between peers, but has largely neglected top-down contagion and the boundary conditions thereof (Gino et al., 2009a,b). Our research indicates that an individual who has higher moral identity levels is less likely to follow their leader’s UPB. This is because an individual who has a higher level of moral identity tends to have higher internal moral standards. When they observe their leaders engaged in UPB, the willingness to follow will be reduced in consideration of the moral character of such behavior, which is different from the individual’s internal moral standards (Hardy et al., 2010). Highlighting the role of moral identify therefore enriches and expands the existing research by considering personal moral traits as the boundary conditions of the downstream contagion of UPB.

Finally, our work also extends the unethical behavior literatures by investigating the contagion of UPB in the workplace. Although other researchers have noticed the contagion of unethical behavior in organizations and have conducted some experimental studies accordingly (Treviño et al., 2014), very little has been done in terms of exploring the issue in the context of real organizations. The findings of our study have stronger external validity than those of prior studies, and can be more easily and directly interpreted for use in organizations.

### Practical Implications

Our study also offers several practical contributions. Given that our findings revealed that a leader’s UPB can prompt employees to follow similar actions, organizations should regulate leaders’ behavior and thereby prevent the spread of UPB within firms. In addition, organizations can establish standards for acceptable behavior by formulating policies that punish LUPB. Furthermore, whereas organizational members are generally encouraged to identify with their leader to create better outcomes (Wing et al., 2018), our results indicate that managers should view employees’ leader identification dialectically. That is, employees should be guided to recognize the morality of leaders’ behavior rather than to blindly follow it. Finally, our research indicates that an employee’s moral identity can deter them from following a leader’s UPB. Given this effect of self-control, organizations should strengthen the moral consciousness of their employees and create an ethical organizational norm.

### Limitations and Future Directions

There are certain limitations in our research that should be noted. First, although we used multisource time-lagged data and our theory supports the direction of the relationships between LUPB and EUPB, the reverse causality of the relationships we suggest is possible. It would be interesting if future studies were to explore the contagion of UPB from employees to leaders. Secondly, we used the willingness scale to measure participants’ UPB, and while adopting this scale to measure UPB is consistent with the definition of the behavior emphasizing an intention to benefit organizations (Umphress and Bingham, 2011), and although the scale has been widely used as a substitute for actual behavior (Miao et al., 2013; Kalshoven et al., 2016; Ghosh, 2017), future research should explore whether measuring actual UPB might lead to different results from those obtained through the present study. Thirdly, although recent research has theoretically explained that LUPB can provoke subordinates to perform similar UPB, it is possible that an unethical organizational culture could also cause LUPB and EUPB to be related, as an individual’s unethical behavior decisions might be influenced by environmental characteristics (Kish-Gephart et al., 2010). Future studies should therefore control for the effect of organizational culture. Finally, in this paper, we theoretically explained the contagion mechanism from LUPB to EUPB, but we did not empirically test this mechanism. Hence, subsequent studies might analyze the specific psychological mechanism through which LUPB relates to EUPB.

## Conclusion

This research proposed that supervisors’ UPB would motivate subordinates to some extent to engage in UPB. Through a field study based in China, we found strong support for our hypotheses: higher LUPB motivated greater EUPB to some extent, and this effect was stronger when the individual had higher leader identification and lower moral identity levels. Our results extend research regarding UPB by highlighting the contagion mechanism from leaders to followers.

## Ethics Statement

This study was reviewed and approved by the Internal Review Board of School of Management, Guangdong University of Technology. All participants gave oral consent after having the purpose of the study described by the researchers.

## Author Contributions

YZ and BH conceived and designed the study. YZ collected and analyzed the data. YZ and XS interpreted the data and drafted the manuscript. BH, YZ, and XS reviewed and edited the manuscript. XS administered the project.

## Conflict of Interest Statement

The authors declare that the research was conducted in the absence of any commercial or financial relationships that could be construed as a potential conflict of interest.
